# Polypharmacy: droxidopa to treat neurogenic orthostatic hypotension in a patient with Parkinson disease and type 2 diabetes mellitus

**DOI:** 10.1007/s10286-017-0435-5

**Published:** 2017-07-03

**Authors:** Steven Vernino, Daniel Claassen

**Affiliations:** 1 Department of Neurology and Neurotherapeutics, UT Southwestern, Dallas, TX USA; 2 Department of Neurology, University of Southern California, Los Angeles, CA USA

**Keywords:** Type 2 diabetes mellitus, Parkinson disease, Neurogenic orthostatic hypotension, Droxidopa

## Challenge question

In patients with polypharmacy, what medications may limit the potential use of droxidopa?

## Case presentation

Mrs. K is a 78-year-old woman with Parkinson disease (PD) for which she is receiving carbidopa/levodopa 25/100 mg three times daily (TID). She also has a history of depression for which she takes duloxetine 30 mg twice daily (BID), type 2 diabetes mellitus for which she takes metformin 500 mg TID, and hypertension for which she takes enalapril 5 mg BID.

Recently, she has been experiencing dizziness and lightheadedness along with occasional falls. During a visit to her neurologist, her blood pressure (BP) in the supine position was 138/70 mmHg, and it dropped to 95/60 mmHg in the standing position, with no significant increase in heart rate (HR) upon standing. The patient was thus diagnosed with neurogenic orthostatic hypotension (nOH). Her neurologist considered starting droxidopa, although he was concerned because droxidopa should be used with caution in patients who are taking norepinephrine transporter (NET) receptor-blocking medications. These are commonly used for the treatment of depression in PD, and include atomoxetine, duloxetine, bupropion, or venlafaxine. As droxidopa increases plasma norepinephrine, the combination of these two medications could have synergistic effects on this patient’s BP. Previous work has shown this synergistic effect with atomoxetine (a NET receptor blocker) and yohimbine (an alpha-2 adrenergic antagonist), which resulted in increased BP [3].

In this case, her neurologist decided that droxidopa was the best medication to treat her symptomatic nOH. To avoid potential synergistic problems between duloxetine and droxidopa, her neurologist transitioned the patient from duloxetine to sertraline, a pure serotonin reuptake inhibitor. Alternatively, the patient could have been prescribed a lower dose of droxidopa and to have been closely monitored. Her neurologist also considered the possibility of using midodrine instead of droxidopa to avoid potential interactions with the NET receptor-blocking medication [4]. Midodrine should be administered with caution in patients with hepatic dysfunction, and it is contraindicated in acute renal failure, urinary retention, pheochromocytoma, and thyrotoxicosis [2]. None of these conditions were present in this patient.

## Expert commentary (Dr. Vernino)

Many patients with PD and nOH also have significant supine hypertension [1, 5]. Additionally, many patients with autonomic failure also have “reverse dipping,” a situation where the BP increases during the night and drops in the morning (unlike the normal circadian pattern of BP). If sleeping with the head of the bed elevated 30–45° is not sufficient to alleviate supine nocturnal hypertension, the use of a short-acting antihypertensive medication such as an angiotensin-converting enzyme (ACE) inhibitor, angiotensin receptor blocker (ARB), or topical nitrate at bedtime might be considered.

## Expert commentary (Dr. Claassen)

For a patient such as Mrs. K, my clinical preference would be to evaluate the role and efficacy of the NET medication, change to a selective serotonin reuptake inhibitor, and then titrate with droxidopa. Furthermore, the patient is taking enalapril, an ACE inhibitor, for BP control. This medication can be administered at bedtime, which, in addition to sleeping with the head of the bed elevated 30–45°, can help to reduce supine hypertension.

## Case continuation

To treat this patient’s symptomatic nOH, she was started on 100 mg droxidopa on a modified TID schedule (taken before arising from bed in the morning, at midday, and at least 3–4 h prior to bedtime) and uptitrated by 100 mg TID every 24–48 h until nOH symptomatic relief or the maximum dose of 600 mg TID was achieved. In addition, to avoid synergistic effects on BP, the patient was switched from duloxetine to sertraline (a pure serotonin reuptake inhibitor) 50 mg taken orally once daily (QD). The patient also was instructed to reduce the dosage of enalapril to 5 mg QD at bedtime.

After achieving a steady dose of droxidopa at 600 mg on the modified TID schedule, Mrs. K reported that she was experiencing significantly fewer occurrences of dizziness and had not experienced any falls for the past 2 months. One hour after taking her noon dose of droxidopa, her BP was 135/80 mmHg while seated and 99/65 mmHg upon standing for 3 min.

## Expert commentary (Dr. Vernino)

This patient achieved a good clinical response with the maximum recommended dose of droxidopa, 600 mg, TID. In patients with neurodegenerative disorders associated with nOH (like PD), droxidopa must be titrated up to the maximum recommended dose or until the occurrence of side effects before considering the patient’s response to be treatment failure. Also, because the metabolism of droxidopa depends on dopa decarboxylase, changes in the dose of carbidopa may require adjustment of the dose of droxidopa. For example, if carbidopa/levodopa were to be discontinued, the physician should monitor for supine hypertension and consider reducing the dose of droxidopa.
